# Perspectives on TB patients’ care and support: a qualitative study conducted in Accra Metropolis, Ghana

**DOI:** 10.1186/s12992-019-0459-9

**Published:** 2019-03-05

**Authors:** Faustina Twumwaa Gyimah, Phyllis Dako-Gyeke

**Affiliations:** Department of Social and Behavioural Sciences, School of Public Health, College of Health Sciences, University of Ghana, P.O. Box LG 13, Accra, Ghana

**Keywords:** Tuberculosis, Direct observed treatment short course, Patient-related barriers, Health system-related challenges, Coping strategies, Phenomenology

## Abstract

**Background:**

Tuberculosis (TB) was declared a global emergency in 1993 by the World Health Organization (WHO). Despite available interventions initiated by the WHO and some countries, the disease remains a key public health problem. The rates of TB infection and its associated burden is unevenly distributed across the globe with greater severity in low-to-middle income countries. This paper therefore explored the experiences of TB patients and health care providers pertaining to patients’ care and support during treatment, in the Accra Metropolis of Ghana.

**Methods:**

A qualitative approach using phenomenology was employed to explore participants’ experiences. Maximum variation sampling, a type of purposive sampling was employed in selecting participants who exhibit a wide range of behaviours and experiences**.** Thirty (30) In-depth Interviews and three (3) Key Informant Interviews were conducted in selected facilities within a period of three months in 2018. The data was audio-recorded, transcribed, and transported into Nvivo version 11, for data management and coding. Content analysis of data was carried out for the generation of themes.

**Results:**

The findings revealed that good knowledge of TB treatment practices did not spontaneously shape perceptions towards treatment. Factors including prevailing cultural beliefs, physical and psychological stress, consequences of patient’s interrupted labour and health system challenges were hindrances in caring for TB patients. Physical, mental and spiritual mechanisms were adopted to cope with challenges.

**Conclusion:**

Personal patient-related challenges and health system bottlenecks were major influencing factors in providing care and support to TB clients. The National Tuberculosis control Program (NTP) of Ghana should adopt measures and provide the required financial, infrastructural and human resources for the augmentation of patients’ treatment.

**Electronic supplementary material:**

The online version of this article (10.1186/s12992-019-0459-9) contains supplementary material, which is available to authorized users.

## Background

Despite available interventions initiated by the World Health Organization (WHO) and many countries, Tuberculosis (TB) remains a key public health problem worldwide [1, 2]. Globally, about one-third of humanity is infected with the TB bacterium although a greater proportion of the population shows no symptoms. About 10.4 million TB incidence and 1.7 million deaths from the disease were recorded in 2016 [3].

Africa is the second highest TB endemic continent in the world. The continent forms about 11% of the population of the world, however, it host about one-third of the global burden of TB incidence and 34% of related deaths [4]. About three million individuals with TB remain undiagnosed and untreated in Africa [5]. In Ghana, the second national TB survey conducted revealed a national prevalence of 290 given a 100,000 population. This figure is about three times higher than the estimated 92 per 100,000 by the WHO [6]. The burden of TB in Ghana is unevenly distributed, and relatively prevalent among urban settlers owing to the challenges of contemporary urbanization such as high population density, poorly planned housing, and poor sanitation in developing countries which facilitate transmission of TB from person to person [7].

The WHO recommended Direct Observed Treatment Short course (DOTS) as an effective strategy for TB control globally. Provision of standard treatment with supervision and patient support is one of the five key elements of DOTS [8]. DOTS has been implemented by the National Tuberculosis control Program (NTP) of Ghana towards the enhancement of TB case detection, supervision of drug intake as well as monitoring the treatment process. DOTS regimens involve the intake of fixed-dose combination tablets. The treatment is divided into two folds including Intensive phase (for newly diagnosed patients) and the Continuous phase (patients who undergo the intensive phase) [9]. In Ghana, patients enrolled in TB treatment, face challenges due to the mode of delivery of DOTS, which requires a frequent visit to the facility. Accessing treatment serves as a major stressor to patients, considering the fact that they have to visit DOTS centres for an extended duration, mostly not less than six months [10]. These challenges can be threatening to the cognitive, emotional, physical as well as the social well-being of patients [11]. During the continuous phase of treatment, patients are seen weekly to monthly in the facility and treatment supporters are required to provide DOT for them at home. Poor adherence and incompletion of treatment have also been found among clients supervised by treatment supporters, mostly family members [12, 13].

In 2015, the United Nations (UN) adopted the Sustainable Development Goals (SDGs), attainable by 2030 with the objective to end epidemics such as tuberculosis. Furthermore, the End TB Strategy targets 95% reduction of deaths due to TB, 90% reduction of TB incidence as well as eradication of all catastrophic costs incurred by affected families between 2015 and 2035. The WHO TB control guidelines updated in 2017, further indicated that when DOT is administered simultaneously with other treatment adherence interventions (e.g. material and psychological support, patient and staff education), treatment outcomes improve significantly juxtaposing with DOT alone [14]. Also anecdotal evidence gathered indicates that the NTP of Ghana is challenged with the implementation of DOTS guidelines, especially within urban settings. Therefore, our study explored knowledge and perspectives on DOTS practice from patients and health workers point of view. We further examined challenges pertaining to the provision of care and support for TB patients, as well as coping strategies that are employed.

## Methods

### Study design and population sample

#### Study design and theoretical background

A cross-sectional study using a qualitative research approach specifically, descriptive phenomenology was utilized. The theoretical underpinning of phenomenology was developed by Edmund Husserl, who postulated that the scientific method, utilized for the study of physical phenomena, was incongruous for the study of human thought and experiences [15]. Using this as a basis, a phenomenological approach recognizes that only individuals who have encountered a phenomenon are able to describe it as a lived experience [16]. Thus, the reality is seen through the meaning that people give to their personal experiences. Utilizing phenomenology in this study was relevant in allowing the researchers’ exploration and description of the subjective views of TB patients’ and their health care providers’ views on DOTS; based on their own lived experiences. The approach also provided a broader platform for assessing the challenges encountered while undertaking treatment.

The study took place in the Accra Metropolis located within the Greater Accra Region of Ghana. Accra Metropolis is highly dense with a population of 1,665,086, females consisting of about 51.9% while males formed 48.1% [17]. Accra metropolis is characterized by diverse administrative, social and economic activities. The NTP of Ghana revealed a high TB incidence in the region with a notification rate of 66 per 100,000 population. This notification rate was greater than the national average recorded in the year [6].

### Recruitment of participants

In this study, 30 TB patients and 3 key informants were selected. The sample size was reached at considering the accepted benchmark of 20 participants required to reach data saturation in a phenomenological study [18]. Medically diagnosed TB Patients registered on the DOTS in the year 2017 or 2018 were eligible for recruitment into the study. Only patients aged 18 years and above, who had been on DOTS for at least two weeks, were selected. This is the age limit for obtaining informed consent under the 1992 Constitution of Ghana [19]. Health care providers (TB coordinators) with at least two years of work experience in DOTS services qualified as key informants for this study.

TB patients and key informants were selected using maximum variation sampling technique which allowed for selecting participants who exhibited a wide range of experiences which was significant in gaining greater insights [18]. Eligible participants were contacted by the coordinators within the selected facilities either in person or through a telephone call. These clients were informed of the study and given the chance to make a decision to willingly participate. Three of the clients contacted however declined the request to participate. The study started from February and ended in July 2018. The data collection period spanned from April to June 2018.

### Data collection techniques and tools

In-depth interview (IDI) which is the primary qualitative data collection method in phenomenology was used [20]. IDIs were conducted at convenient times after patients had visited the facility for their treatment. Key Informant Interviews (KIIs) were also conducted with TB coordinators in the sampled facilities. Informed consent was sought from the key informant and the patients before the audio-recorded interviews were conducted. In addition, confidentiality was assured. The principal investigator together with trained research assistants conducted all the interviews in English and two other local languages (Twi and Ga). Each interview lasted for an average of 30 min.

Two semi-structured interview guides were developed for IDIs and KIIs. The frequent interaction with the NTP team, health care workers at the DOTS centres and researcher’s own observations shaped these questions. Further extensive consultation with two health research specialists with qualitative research expertise was made to fine-tune the questions. The interview guides were then pre-tested and more probe items added to elicit adequate information from study participants. The patients’ interview guide was preceded with socio-demographic data, information on the phase of treatment and adherence to treatment. The general life history of the patients involving their daily activities prior to the onset of the disease, the discovery of TB as well as their reaction when informed of their TB status, were explored. In addition, the guide also explored thematic areas such as patients’ knowledge on TB and DOTS practices, perceptions on DOTS, psychological and social factors influencing treatment compliance as well as the challenges encountered when undergoing treatment and coping strategies. The second guide focused on key informant’s knowledge on TB case detection and diagnosis, perceptions on DOTS, and TB treatment regimen. More so, the barriers to the implementation of DOTS were also explored. The tools were developed and pilot-tested after which more probe questions were added which elicited detailed responses from the participants. Field notes covering initial interviewee’s reactions to the interview, and relevant observations such as the demeanour of the respondent were recorded promptly after interviews. Data gathered were stored with limited access to the research team. The measures for ensuring qualitative trustworthiness according to Lincoln and Guba were applied in this study. The approach employed includes a prolonged engagement between the researchers and the researched followed by member checking used to ensure credibility [21]. The key informants were also contacted after the interviews to verify certain responses given earlier.

### Data analysis

The audiotaped interviews were then transcribed verbatim. The transcripts were read all over again and grammatical errors were edited before they were imported into NVivo 11 software for analysis. A thematic analysis was used employing both deductive and inductive analysis [15]. A codebook was created based on the objectives of the study and the subject areas explored during the interviews. Each transcript was opened in the NVivo software and line-by-line reading and coding into nodes of all the statements were done. The coding was reviewed, where some nodes were rearranged and others merged to develop themes. As coding continued, codebook developed initially was revised. Afterwards major and sub-themes were identified and the table of themes was exported into word for further interpretation of the data. Also, each node was exported back into word for easy reading and selection of the best quotes which were presented in the results section of the work.

## Results

The findings are presented under the ensuing headings; Characteristics of study participants, Patients’ perspectives on DOTS, Patient-related challenges, Health system barriers and Coping strategies Adopted.

### Characteristics of participants

The age range of the TB clients was between 19 and 68 years, whereas that of the key informants ranged between 35 and 59 years. The Key informants had years of work experience ranging from 8 to about 35 years. Most of these clients were employed in diverse jobs before the onset of the disease. More so, patients who were in either the intensive or continuous phases of treatment were included. This provides a blend of experiences at different periods during the treatment (See Additional file 1 for details of Characteristics of participants).

### Patients’ perspective on DOTS

The findings give an understanding of the relevance of DOTS from the participants’ perspective. Many of the patients expressed favourable perspectives on the purpose of DOTS. The participants described the mode of TB treatment as significant for enhancing adherence, monitoring progress and a form of support for clients. The participants stated revealed that their favourable descriptions of DOTS emanated from the experience of support from treatment providers in overcoming risky behaviours such as smoking and excessive intake of alcohol, they engaged in earlier which hindered adherence to anti-TB medication. For instance, one participant mentioned that “*At the initial stage, we were educated not to smoke, drink alcohol, and the need to eat well…Coming here often allows them [health workers] to monitor how far one is progressing when the tests are done. They are able to know if you are complying or not”* ***(38y/o, Male, Facility 3).*** Participant’s again held a positive perception of drug dosage and frequency of intake. A male participant who believed in the curability of TB and the efficacy of the anti-TB drugs, mentioned that *“Taking 4 tablets a day for me is not a bother. Even if it is ten tablets I will take so long as I have been assured that I will be healed…”* ***(29y/o, Male, Facility 1).*** Thus, to such participants, assurance of the efficacy of anti-TB drugs was a key contributing factor in compliance to treatment. Furthermore, DOTS was described as beneficial to all the participants due to positive outcomes such as reduced symptoms and recovery of strength. A male participant shared his perception of drug adherence. He stated that “*Physically, they [adherent clients] are fine and I am even a testimony. When I came here earlier, I was very lean, but within a short period of time, the doctors and nurses were testifying that I have become very fine”* ***(40y/o, Male, Facility 3).*** Some participants reported emotions of despair and worry at the inception of treatment due to experiences of threatening symptoms and long duration for treatment. These clients however, regained enthusiasm due to the efficacy of drugs and treatment outcome.This is shown in the quote below:

*“I was shocked when I was told I would be taking the drugs for that long [six months] but now I am ok. Once I will get healed, I do not care if the duration is even one or two years”* ***(29y/o, Male, Facility 1).***

### Negative perspectives on TB treatment

On the contrary, participants described the anti-TB drug size and dosage as unfriendly. Drugs administered to clients in the intensive phase of treatment were classified as bigger in size and difficult to swallow. Though drugs administered during the continuous phase of treatment were more favourable in terms of ingestion, patients’ perceived it as less effective compared with intensive phase drug combination. According to the patients, the less effectiveness of such drugs led to the reoccurrences of previous symptoms as shown below:

“*The first set of drugs for the intensive phase of treatment was very effective and good. Almost all the symptoms I used to have had vanished…but when they switched to the second type of the drugs, I realized that the symptoms were re-occurring again” (****42y/o, Male, Facility 3)****.*

Furthermore, some of the respondents had negative perceptions about the duration of DOTS. Despite the counselling and health education received on the efficacy of the drugs, clients maintained their opinion about the duration of the treatment as very long. Clients who had high education and those with health background perceived regular visit to facilities for supervision to be unnecessary. For instance, one patient mentioned *“…I don’t think I need anyone to tell me to come for my drugs. If the person is well informed [about treatment] he/she should be exempted”* ***(42y/o, Male, Facility 1).*** Relatedly, another client said that *“To me, this duration should be shortened and a stronger drug should be given to us”* ***(34y/o, Male, Facility 1).***

### Patient-related challenges to TB patients’ care and support

Participants of the study experienced physical and psychological challenges in adhering to DOTS within health facilities. Major themes that emerged included physical and psychological stress as well as consequence of interruption of labour.

### Physical and psychological Stress

Factors such as old age, commuting to health facility on foot on a regular basis and pregnancy-related challenges accounted for the physical stress experienced by clients. Clients interviewed who were either older adults or former vehicle drivers, faced the burden of frequently joining ‘trotro’ (public transport) to be attended to by health workers in their respective DOTS centres. Consequently, one patient mentioned that *“…coming here [health facility] is very stressful”* ***(68y/o, Male, Facility 2),*** whilst another said that *“I get stressed coming here, especially when I am walking”* ***(42y/o, Female, Facility 3).*** Commuting to DOTS centres was a usual activity for most of the participants due to inability to afford alternative means of transportation. Clients who were often weak at the initial stage of treatment were also burdened with this stress in accessing treatment daily. Similarly, a pregnant woman lamented on the daily struggles she encountered in accessing treatment in a health facility. She explained that *“Every day, I sit in a vehicle. Sometimes I do vomit when I take the drugs [in the facility] because of hunger but if I were home, I take the drug and eat there and then...So it was stressful”* ***(26y/o, Female, Facility 1).*** The need to report early to the facility each day for treatment was a challenge due to the stress in travelling to the facility owing to the poor road network and the long traffic jam evident in the study area. Moreover, the pregnant woman who is unable to withstand hunger takes the drugs and had to wait an hour before eating.

In addition, clients who had the opportunity to be transferred to facilities easily accessible to them were unwilling to comply with this directive due to perceived societal stigma. This accounted for some stress due to the need to wake up earlier and walk or drive longer to comply with treatment. Others resorted to hiding and sneaking back and forth the facility when attention was not drawn on them. For instance, one client mentioned that *“I always hide and make sure no one sees me. I think the DOTS centre should be situated away from this place, where lots of people are not there”* ***(34y/o, Male, Facility 1).*** Such experiences explain the misconceptions held around the disease. The clients feared of being withdrawn from by others. Such a threat is likely to pose psychological stress on those who experience them.

### The consequence of interrupted labor

It was identified that all the clients had to quit their respective jobs at the commencement of treatment. In addition, the majority of the participants were artisans and drivers who depended on physical strength to work. Thus, the physical weakness experienced incapacitated patients to continue working. This implied an interruption of career and/or seizure of the source of livelihood for clients. Respondents revealed the challenge of inadequate material support owing to their inability to work. Food and transportation burden was inevitable which had an influence on their intention to comply with treatment as shown below:

*“…We [all patients on DOTS] have been told to eat well, but it is difficult to eat three times because of the money issue. This can even make it difficult for one to even comply with the treatment since once you take the drug you have to eat well”* ***(38y/o, Male, Facility 3)*** *.*

In addition, the burden of caring for clients and their families was placed on their spouses, other family members, and sometimes health care providers. A participant revealed the financial burden placed on his spouse due to his inability to work. He stated that *“I am not working and all the pressure [financial demands] is on my wife including the payment of school fees of the children and feeding expenses”.* ***(41y/o, Male, Facility 3).*** Relatedly, health workers also shared the burden in providing support and care for TB patients. All the health workers interviewed reported the kinds of material support they provide for their clients in the form of food, money for transportation and personal up-keep, among others.

Participants, especially males who depended on others for financial support reported unfavourable experiences from community members. Culturally, it was expected of males to engage in productive roles. Thus, anyone who was unable to meet such expectations was considered irresponsible. Such individuals became victims of public ridicule. A participant, for instance, stated that *“…because I am not working sometimes I feel people are looking at me even when I am passing by”* ***(41y/o, Male, facility 3).***

### Health system barriers to TB patients’ care and support

#### Inadequate health education and counseling services

Participants reported that some health education on TB and its treatment was made available to TB patients and their primary contacts when clients enrol on DOTS. Patients demonstrated good knowledge of TB, particularly regarding predominant risk factors, symptoms, transmission, and prevention of the disease. However, there were still some misconceptions among the patients. It was revealed that participants attributed the cause of TB a supernatural source. For example, one client attributed the cause of her illness to the work of a spiritual enemy with the aim of attacking her family. She stated that *“… my mum was affected [with TB] and my brother and me. I can say that ours is spiritual, it is not the normal TB because ours was only within our family…We were just being attacked…”* ***(26 y/o, Female, Facility 1).*** It was observed that the health education and counselling services provided were inadequate to demystify the prevailing beliefs held by patients and their contacts.

In the light of such misconceptions, key informants explained the health-seeking behaviours of community members and its consequence on the prognosis of the disease. Late reporting to the facility for treatment was one of such consequence. To a high extent, late reporting culminated in death in some cases. A key informant stated that *“Some [patients] believe they might die when they take the drugs. As a result, they show up for medical care for TB late, the drug might not work on them at that time and they die. Others think TB is a spiritual sickness and they see no reason why they should take medicine for something that is spiritual”* ***(KII, 35y/o, Female, Facility 1).***

In addition, not only do these misconceptions hinder prompt enrollment unto treatment but also leads to interruption of treatment. Patients wrongly attributed the intake of anti-TB medication to the threatening symptoms they experienced, such as coughing of blood. These misinterpretations influenced the decisions of clients to seek treatment from herbal and spiritual centres, which they believed to be more effective in the treatment of TB. For example, a patient stated that *“I took it [anti-TB drugs] for two months...Then I went for herbal treatment [for one month]”* ***(40y/o, Male, Facility 2).*** Relatedly, it was also reported that family members influenced clients to default treatment because of their lack of knowledge about the treatment.

In relation to the provision of counselling services, the results show a gap in the quality and availability of the service to clients and their contacts. The lack of psychologists to provide professional counselling to clients especially difficult clients (patients who miss treatment frequently), was observed. Patients expressed the stigma they experienced from their spouse, family, and employees without prompt counselling support. A client, for instance, stated that *“My family members started avoiding me because they all thought it [TB] was deadly and that I was not going to make it. My wife even packed her things and left with the kids to their family house…Even if I ask an employee to do something for me, they give me excuses because they do not want to get close to me. They will say TB is more deadly than HIV”* ***(42y/o, Male, Facility 3).*** These experiences left patients confused, hopeless, which affected their behaviour. However, there was no readily available counselling service for such clients.

#### Healthcare workers challenges

It was observed that the healthcare providers lacked protective gears for infection prevention as they provided treatment for the patients. Health care providers were required to monitor the swallowing of anti-TB drugs by clients. The health workers usually get closer to the clients for them to feel accepted and catered for. However, without the necessary protective wares, caring for the patients becomes threatening to the safety of the staff who attend to especially patients in the intensive phase of treatment. Thus, the psychological wellbeing of health workers treating TB clients was also affected. In some cases, the relationship between patients and health workers was strained out of the fear of infection experienced by health workers. A client lamented on her encounter with a health worker due to her *inability to observe the required coughing etiquette while in at the DOTS centre. She stated that “I cough but I use my hand to cover my mouth but the way she [health worker] shouted at me, that made me quarrel with her. I told her not to talk to patients like that”* ***(26y/o, Female, Facility 1).***

In addition, there is low staff strength at DOTS centres. This indicates an increase in the workload of staff. Activities such as home verification and home support visits were hindered. The staff who are overburdened with high workload also face the challenge of lack of physical motivation. More so, health care providers reported the challenge of inadequate funds available to allow for the frequent home visits of TB patients.

#### Coping strategies adopted

The main themes that emerged include, Physical as well as mental and spiritual mechanisms.

#### Physical coping mechanisms

Patients described behaviour modification strategies they adopted to overcome challenges experienced in accessing TB treatment. Participants who lived far away from the facility resorted to travelling at dawn. One client said, *“I come all the way from Swedru [a town outside the study area] so I have to wake up around 3 am and pick the first car to this place****”(40y/o, Male, Facility 2).*** This indicates that the high TB prevalence as measured in Greater Accra Region may be imported cases from other regions. Clients also modified their behaviours by refraining from smoking and the intake of alcohol. Others broke off relationships to avoid risky behaviours, thus enabling them to continue with the treatment. A client stated that; “*If I should be moving with my friends I will start drinking again. It’s been a while since I met with them”* ***(48y/o, Male, Facility 3*)**.

In addition, patients who perceived health facility DOT as frustrating resorted to self-administration of treatment as a favourable mechanism. SAT was common among the aged, critically ill, and clients who were unavailable for health facility-based DOT due to travel schedules. This mode of treatment was supervised by health workers. A client stated that “*They told me that when I take it for a week, I have to bring the pack for them to see. Then it proceeded to two weeks, three weeks and then a month” (****26y/o, Female, Facility 1).*** Furthermore, swallowing anti-TB drugs with cold water and breaking tablets also enhanced the ingestion of drugs.

Again, relocation to a different community was a means a client utilized in the face of community stigma. A couple who faced this challenge relocated into a family house, farther away from DOT centre. Though this act had transportation cost implication on accessing regular treatment, it was an intermittent agency to avoid communal stigma.

In addition, to some clients marriage provided financial, emotional and physical support to some TB client. The performance of reproductive roles such as house chores by male partners in support of their spouse was observed. A patient who was married pointed out that, *“Throughout my sickness, had it not been for my husband...I don’t think I would be alive by now”* ***(38y/o, Female, Facility).*** Similarly, support for TB patients was also obtained from other relatives, friends and community members. Other clients coped with the treatment burden through alternative source of incomes. Furthermore, the readily available assistance from physicians enhanced management of adverse effects of drug intake.

#### Mental and spiritual coping mechanisms

Developing a positive mindset towards treatment was a resource resorted to by patients despite the adverse physical, financial, societal and drug-related burdens they experienced. For instance, a patient who was abandoned by his family members developed a mental coping resource to overcome his challenges. He stated that *“So because of the stigma, I have been able to psyche myself about how people relate to me. Now I understand everybody. As a result, I am fine now”* ***(42y/o, Male, Facility 3).*** In other cases, non-disclosure of TB and denial of the disease were adopted as supported with the quotes below:

“*I did not want to tell them[friends] because my mother sells food and if I should tell my friends, maybe my mother’s market will go down”****(29y/o, Male, Facility 1***

“*As for me, I knew I had not gotten any TB so I was not scared…I do not worry because I believe I do not have the illness”* ***(63y/o, Male, Facility 1).***

On the other hand, a spiritual coping mechanism was also reported, such as the belief in the existence and power of God in healing diseases. Some patients relied on this spiritual belief to cope with challenges such as deteriorating health, fear of the death, and inability to comply with treatment. For instance, a client pointed out the source of her strength in coping with treatment demands. She stated that *“…whatever I ask God that is what he will give to me…I always ask him [God] to strengthen me…I combine house chores with pregnancy and TB treatment. God helped me”* **(26y/o, Female, Facility 2).**

## Discussion

This paper had demonstrated the consequence of TB on the lives of patients in the Ghanaian setting. The use of phenomenology provided a broad approach for identifying the facilitators of and barriers to DOTS. Exploring the lived experience of TB patients revealed the benefits of DOTS practices to all the study participants. These patients who saw symptoms stopping after few weeks on treatment, also benefited from support from health care providers and significant others. Such experiences were expressed as positive attitudes towards the duration of treatment, dosage, and frequency of drug intake. However, despite the good knowledge patients’ had on TB, their perceptions towards treatment were culturally defined. This finding indicates that knowledge of TB treatment practices could have no influence on perceptions on DOTS practices, which is similar to findings in a Pakistani community [22]. Treatment seeking experience of patients was influenced by the broader cultural context of the country. Majority of Ghanaians seek healthcare from non-orthodox outlets such as traditional healers and spiritualists despite the proliferation of orthodox medicine due to globalization [23]. It is also estimated that about two-thirds of healthcare in Ghana is provided by traditional healers. The belief in traditional healing practice highlights patients’ negative perceptions towards DOTS, and treatment defaulting behaviour [24]. Therefore, adherence to TB treatment goes beyond the provision of health education. There is a need for professional counselling services. On the contrary, the findings of the current study reveal the limited availability and gap in quality counselling services required to demystify these prevailing misconceptions. Thus, contributing to the psychological stress experienced by TB patients and healthcare providers themselves. Similarly, another study reported that low and middle-income countries are faced with a short supply of trained psychologists and counsellors [25].

Furthermore, participants reported the financial assistance required from the NTP as inadequate or unavailable (in some instance) in catering for their basic needs whiles on treatment. Patients experienced catastrophic costs due to frequent facility visit, increased food demands and other family financial burden. This finding is similar to that of previous studies [26–32]. Thus, reflecting the 2017 estimate of 2.3 billion US dollars global financial gap required for TB diagnosis and treatment [33]. LMICs are therefore required to adopt measures to domestically mobilize resources for TB patients care and support. This is critical in achieving the 2020 milestone of the End TB strategy specifically by removing catastrophic cost as a consequence of the disease.

The study further revealed that TB patients experience physical stress. Physical stress was experienced due to the difficulty in accessing treatment in health facilities due to poor transportation and road network in Ghana [34]. It was also been noted that the socioeconomic status of patients influence the level of physical stress experienced [35]. This explains why in the current study, TB patients rendered unemployed during the period of treatment reported more stress-related symptoms and challenges. Again, stress-related factors such as age, gender, and place of residence accounted for patients’ intention to default treatment. Client’s decision to continue with the treatment was however enhanced by the support they occasionally received from the staff and treatment supporters. Previous studies show that follow-up actions and DOT at home enhanced treatment compliance among pregnant women [36]. Furthermore, living with psychological stress in the form of anxiety, hopelessness, worry and fear were common experiences peculiar to clients with low self-efficacy and the lack of belief in the curability of the disease. This study has been reported in previous studies in Southern Africa [32, 37]. In the current study, the assurance patients had in the efficacy of anti-TB drugs was a contributing factor for compliance to treatment regardless of experienced side effects.

Through the utilization of a phenomenological approach the physical, mental and spiritual coping agencies adopted were explored. Patients described the behaviour modification measure adopted that has been beneficial in enhancing their compliance with treatment. Patients addicted to alcohol intake and smoking had to break off negative friendships to concentrate on treatment. The study also found SAT as a critical agency in overcoming the financial and physical burden patients experienced due to the daily visit to facilities for treatment. This result is similar to those of previous studies [38, 39] that demonstrated that SAT was commendable when resorting to DOT was difficult. Though the findings of this study show that health care providers were skeptical in adopting this measure, SAT received in conjunction with treatment adherence interventions such as material support, psychological support, and patient education enhanced treatment compliance [14].

To cope with the prevailing TB induced stigma some patients adopted non-disclosure of TB whereas others could deny the symptoms even after a diagnosis of TB. These strategies adopted, however, were problematic. TB patients capitalized on peoples’ ignorance thus placing them at high risk of infection. Non-disclosure of disease could as well be to the disadvantage of patients whereby social support would be unavailable as revealed in a previous study [40]. Denial of TB has also been reported in a previous study in the Western Regions of Ghana where TB patients attributed their symptoms to malaria and other non-stigmatized illnesses [23].

### Limitation of the Study

There was a challenge in finding a suitable location for the execution of the interviews. The difficulty in recruiting participants led to the interviewing of patients in the health facility right after their visit for treatment. Participants selected into the study were also predominately males. This was due to the higher number of males enrolled in DOTS compared to female patients in the region [6]. Again, there was a subjective researcher positionality in the conduction of the study. The selection of the research topic, aims and method were influenced by researcher’s interest in Tropical Disease Research with funding from WHO. These limitations, however, did not influence the quality and credibility of data obtained.

## Conclusion

The psychosocial and economic barriers hindering TB treatment has been revealed. To enhance the robustness of the TB patient’s care and support, the NTP of Ghana should intensify TB awareness creation and adopt pragmatic fundraising measure at the governmental and non-governmental levels to cater for the financial gap created. There is also the need for the NTP to modify health education to target overcoming the socio-cultural barriers observed. Furthermore, the NTP should see to the establishment of Clinical Psychological units within facilities as well as train health workers to bridge the gap in the provision of quality psychological services to clients and health workers. Last but not least, in order to enhance patients’ access to drugs outside assigned facilities, the community/home-based DOT programs in the country should be strengthened and duly utilized since this can reduce travel costs, stress and the spread of TB resulting from patients travelling to seek care.

## Additional file

Additional file 1: Characteristics of Study Participants (DOC 50 kb)

## Abbreviation

DOT: Directly Observed Therapy
DOTS: Direct Observed Treatment Short course
GHS-ERC: Ghana Health Service-Ethical Review Committee IDIs: In-depth Interviews
KIIs: Key Informant Interviews
LMIC: Low and Middle Income Country
NTP: National TB control Programme
SAT: Self-Administration of Treatment
TB: Tuberculosis
WHO: World Health Organization
