# Supplementary material for: Perspectives on TB patients’ care and support: a qualitative study conducted in Accra Metropolis, Ghana
Source: Global Health. 2019 Mar 5;15:19. doi: 10.1186/s12992-019-0459-9 (PMC6402088; doi:10.1186/s12992-019-0459-9)
Supplement: Supplementary file 1 — Characteristics of Study Participants (DOC 50 kb) [file 12992_2019_459_MOESM1_ESM.doc]

Table S1: Characteristics of Study Participants

| **Characteristics of Participants** | **Number of Participants** | | | |
| --- | --- | --- | --- | --- |
|  | **Patients for IDIs** | | **Key Informant** | |
| **Facility Visited by Patient** |  | | |  |
| Achimota Hospital | 10 | 1 | | |
| Kaneshie Policlinic | 10 | 1 | | |
| Greater Accra Regional Hospital | 10 | 1 | | |
| **Sex** |  |  | | |
| Male | 22 |  | | |
| Female | 8 | 3 | | |
| **Age (years)** |  |  | | |
| <20 | 1 |  | | |
| 20-29 | 4 |  | | |
| 30-39 | 6 | 2 | | |
| 40+ | 19 | 1 | | |
| **Educational Level** |  |  | | |
| No Formal Education | 1 |  | | |
| Primary | 2 |  | | |
| JSS/Secondary | 23 |  | | |
| Tertiary | 4 | 3 | | |
|  |  |  | | |
| **Religion** |  |  | | |
| Christianity | 27 | 2 | | |
| Islam | 3 | 1 | | |
| **Occupation** |  |  | | |
| Trader | 7 |  | | |
| Craftsmanship | 6 |  | | |
| Researcher | 1 |  | | |
| Transport Industry | 5 |  | | |
| Farmer/Fisherman | 2 |  | | |
| Unemployed/Student | 3 |  | | |
| Administrator | 6 |  | | |
| Nurse |  | 3 | | |
|  |  |  | | |
| **Marital Status** |  |  | | |
| Single | 5 | 1 | | |
| Married | 20 | 2 | | |
| Divorced | 3 |  | | |
| Widowed | 2 |  | | |
| **Phase of Treatment** |  |  | | |
| Intensive | 11 | | |  |
| Continuous | 19 | | |  |
|  |  | | |  |
